# Direct-to-consumer genetic testing

**DOI:** 10.5808/GI.2019.17.3.e34

**Published:** 2019-09-27

**Authors:** Jong-Won Kim

**Affiliations:** Department of Laboratory Medicine and Genetics, Samsung Medical Center, Sungkyunkwan University School of Medicine, Seoul 06351, Korea

**Keywords:** direct-to-consumer test, DTC, genetic prediction, genetic testing

## Abstract

Direct-to-consumer (DTC) genetic testing is a controversial issue although Korean Government is considering to expand DTC genetic testing. Preventing the exaggeration and abusing of DTC genetic testing is an important task considering the early history of DTC genetic testing in Korea. And the DTC genetic testing performance or method has been rarely reported to the scientific and/or medical community and reliability of DTC genetic testing needs to be assessed. Law enforcement needs to improve these issues. Also principle of transparency needs to be applied.

## Direct-to-Consumer Genetic Testing Definition and Current Situation

Direct-to-consumer (DTC) genetic testing is a method of marketing genetic tests to consumers without the direct involvement of a health care provider [1]. DTC genetic testing in Korea has been introduced since 2017 by the amendment of the Bioethics and safety Act. Through the amendment, 12 phenotypes by 46 genes have been allowed for DTC genetic testing. Those phenotypes include traits of body mass index, cholesterol, blood pressure and so on [2]. Recently, the Korean government introduced a regulatory sandbox that includes DTC genetic tests [3]. Several genetic testing companies received approval for predictive DTC genetic tests for conditions ranging from cancer to chronic diseases. And also, the ministry of Health and Welfare is doing the pilot program of the expanded DTC genetic testing [4]. DTC genetic testing has a lot of issues from accuracies to ethical, legal and social issues (ELSI). I raise the immediate issues relevant to DTC briefly.

## Does DTC Genetic Testing Have Right Test Names?: Relationship with the Promoting Test Name and the Scientific Implication

There is a high chance of exaggerated advertisements and abuses of DTC genetic testing. For example, DRD4*7R allele was reported as associated with novelty seeking [5]. Persons with high “novelty seeking” are described as “impulsive, exploratory, excitable, disorderly and distractible” [6]. But the companies described DRD4 test as detecting creative trait to the people in Korea and promoted especially for children [7]. Novelty seeking trait with DRD4 study is strictly defined by tridimensional personality questionnaire for the genetic study. Novelty seeking seems to share the impression of “creative trait” to the laypeople but when it comes to research area, it is a completely different subject. And from the beginning of the DRD4 genetic studies, they often refer to the antisocial behavior [6]. And the original researchers reported DRD4 was not associated with novelty seeking at 2002 [8]. But even after the report, several companies continued the sales of DRD4 test as searching creativity and DRD4 test was prohibited by law at 2007. Similar cases can be occurred anytime again.

## Is DTC Genetic Testing Reliable?

Most important concern is whether commercial genetic services’ predictive value is sufficient to meet the standards for clinical use. The clinical utility of a genetic test should be an essential criterion for deciding to offer this test to a person or a group of persons [9].

Reliability has been one of the major issues from the beginning even in United States. Dr. Craig Ventor reported the discrepancies of the interpretation of the same individuals between 23andMe and Navigenics [10]. GAO (Government Accountability Office) in United States also investigated inconsistent interpretations among DTC genetic testing companies [11]. And more, Tandy-Connor et al. [12] reported 40% of variants in a variety of genes reported in DTC raw data were false positives in United States. There was a comparison of two persons between two Korean DTC genetic testing companies and the concordance rate is low [13]. It is very difficult to judge which one is the accurate result when the discrepancies happen. Even when their results show the same results, it does not guarantee the results are true.

Establishing reliable prediction models by DTC genetic testing needs advancements under current situation. Currently most DTC genetic testing companies use single nucleotide polymorphisms (SNPs) for predictive models among genome-wide association study (GWAS) results from the relevant literatures. In case of single SNPs for complex disease or trait, its predictive performances would be almost meaningless because odds ratio of most validated SNPs are below 1.5 except few SNPs such as *APOE4* allele to Alzheimer’s Diseases [14]. That is, without counting the other many SNPs, counting only one SNP is not likely to show good performances as company claims. Thus, it comes to use multiple SNPs for predictive models.

In case of using multiple SNPs, Some SNPs come from the study of Korean subjects, but many SNPs depend on the results from Caucasian subjects. Considering the experiences from GWAS studies among Korean subjects, most validated SNPs from Caucasian subjects without studying Korean subjects are likely to be replicated among Korean subjects although *not all of them*. However, when applying to Korean population, the odds ratio of the used SNPs in the predictive model is critical to calculate the relative risk of the individual *consumer*. But the magnitudes of relative risks of the each validated SNPs between Caucasian and Korean subjects do not show same values. Therefore, the established models for each trait need to be validated for Korean patients prospectively at least. But the most company-run or company-plan-to-run traits show lack of these evidences. It is not possible to judge or estimate which company supports better predictive models or result. This situation drives to go to marketing with exaggerations and abuse of tests or price competition rather than competing to improve the quality of prediction.

## Amendment of Law and the Rising of Transparency in DTC Genetic Testing

The current DTC genetic testing needs more objective evidences. If they have a scientific basis from Korean population, even poor performances can have a chance to improve because they know which part needs to be updated or changed. But companies extremely rarely show the method and performance of their predictive model from Korean population.

They demand to the government authorities to allow disease prediction services as DTC genetic testing including cancer, diabetes and heart disease without disclosing their models and performances to the scientific or medical societies. If it is allowed, it will cause the profound confusion or chaos to the consumers, medical institutions, and health insurance system and the huge waste of medical resources. Currently the facility and human resources in DTC genetic testing company are not regulated by law. There is no penalty to their rejection to proficiency test by law. For improving the situation with the above mentioned direction, the legal coercion is inevitable. Amendment of Bioethics and Safety Act or making a law equivalent to Clinical Laboratory Improvement Act (CLIA) in United States for appropriate regulation is a prerequisite step.

The predictive models of DTC genetic testing need the validation before implementation. But practically not all of the tests can be validated or achieved to the certain standard.

If so, disclosing all the relevant information *transparently* for the right choices by the consumers instead can be considered. The relevant information includes the explanation of the whole process of the tests, the references of the method they used, disclosing the implication and limitation of the results, how to deliver the report to consumers etc. And the company should consider the possible outcomes after getting trait(s) risk and provide the report guidelines not to cause any harm or negative effect to the consumers including psychological distress or waste of medical resources.

New genomic technologies and knowledges expand our view and their applications would improve human health. Medical diagnosis and treatment is shifting to genetic based precision medicine. Its progress is strictly guided by evidence-based medicine (EBM). In contrast, if DTC genetic testing with lack of equivalent level of EBM is not regulated, our society will pay a lot.
